# Supplementary material for: Deciphering the evolutionary history of ectoine catabolism, a compatible solute utilized by Vibrio diabolicus as an osmoprotectant and a nutrient source
Source: Appl Environ Microbiol. 2026 Apr 20;92(5):e00409-26. doi: 10.1128/aem.00409-26 (PMC13188858; doi:10.1128/aem.00409-26)
Supplement: Supplemental figures — Fig. S1 to S11. [file aem.00409-26-s0001.pdf]

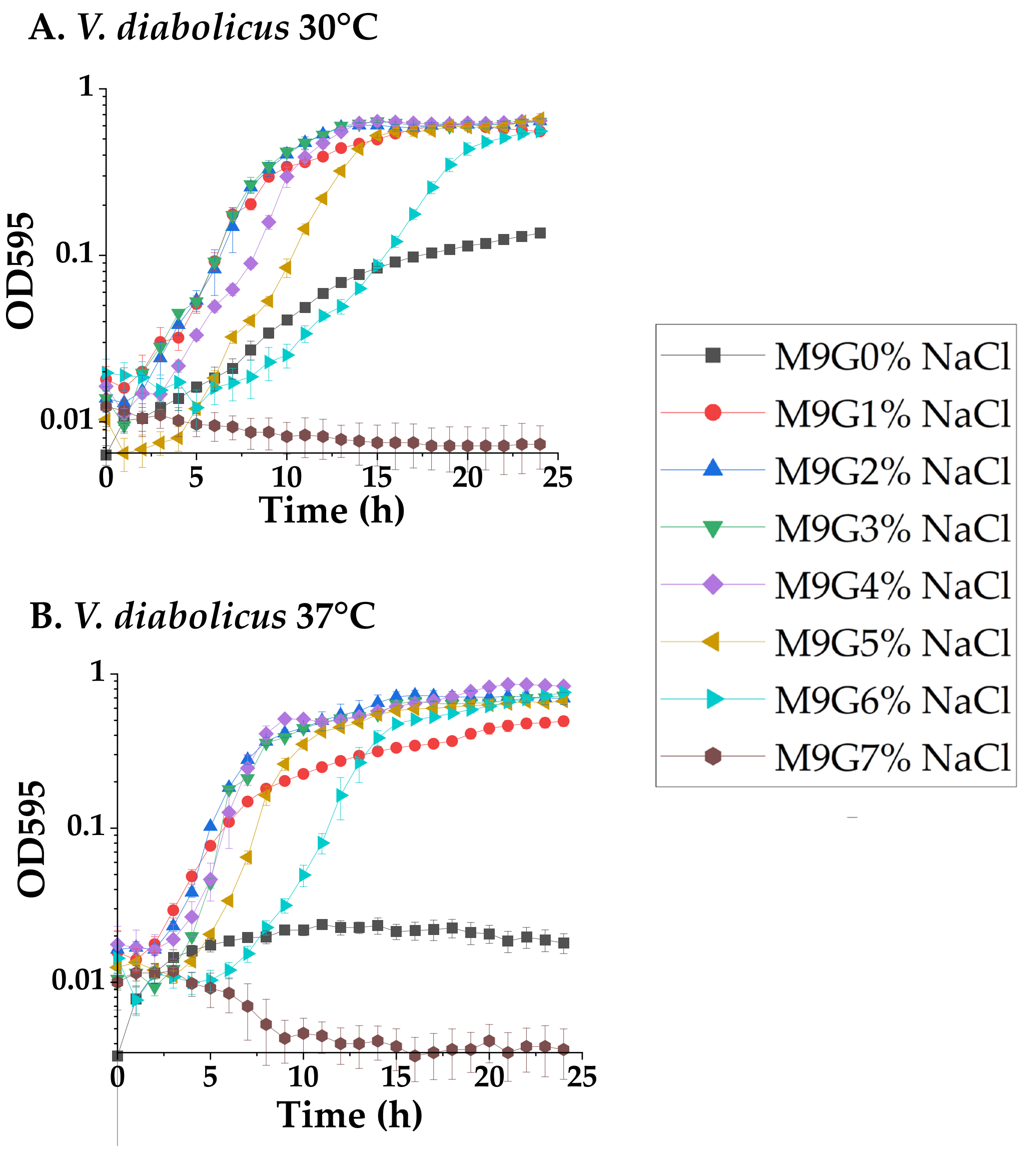

Fig. S1. *Vibrio diabolicus* 3098 NaCl tolerance range and growth at different temperatures. Growth curves of *V. diabolicus* strain 3098 in minimal media (M9) with glucose (M9G) with a final concentration of 0% to 7% NaCl. Growth was measured every hour for 24 h at **A.** 30°C and **B.** 37°C.

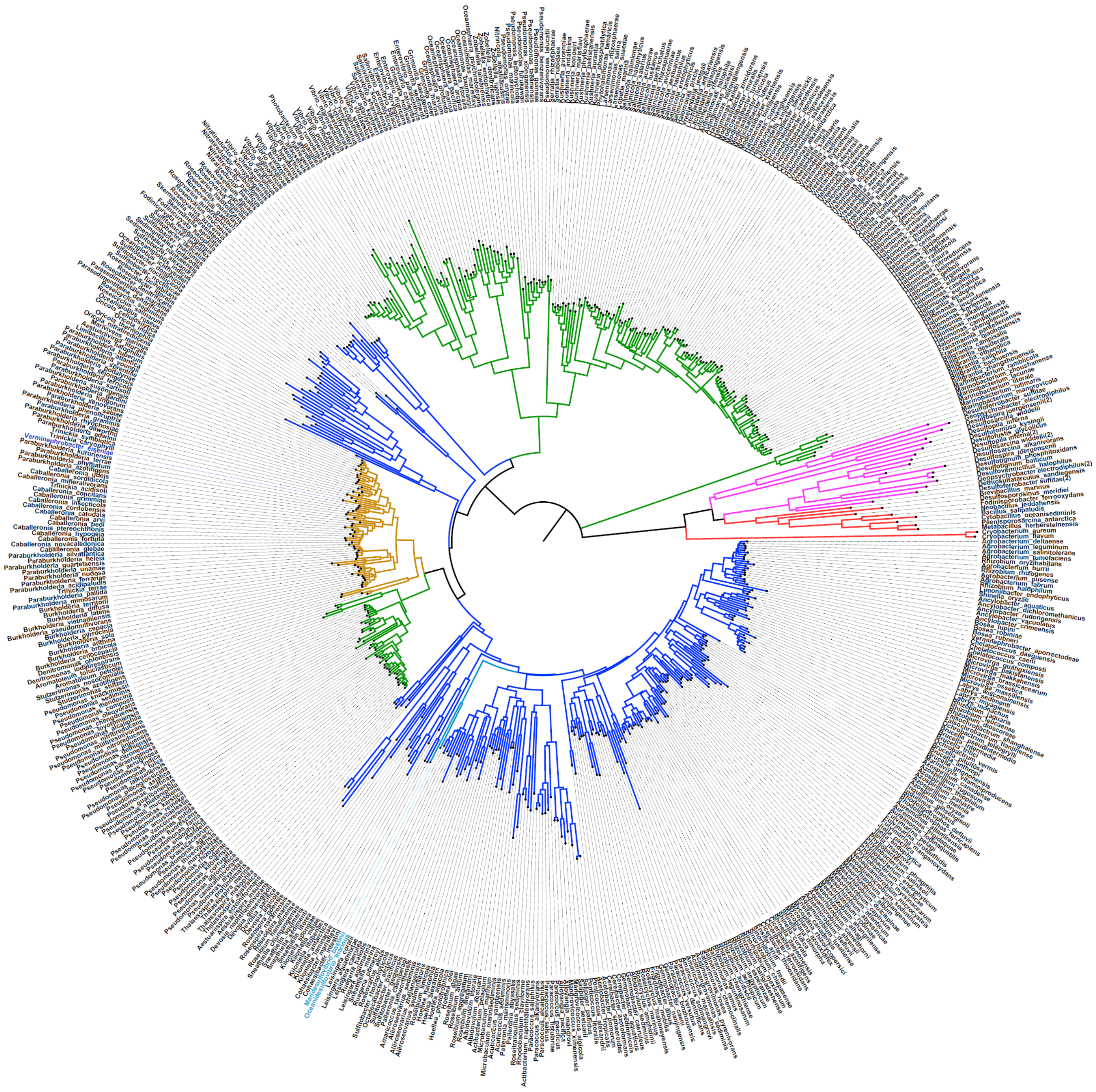

Fig. S2 Labelled Fig. 8. Phylogeny of EutD (DoeA) from 575 taxa. The evolutionary history was inferred using the Neighbor-Joining method with distances computed using the JTT matrix-based method. The complete deletion option was applied resulting in a final data set comprising 378 positions. Evolutionary analyses were conducted in MEGA12 [4] utilizing up to 3 parallel computing threads. Branch coloring is as follows: Green=Gammaproteobacteria, Yellow=*Betaproteobacteria*, Blue=*Alphaproteobacteria*, Aqua=*Deltaproteobacteria*, Red= *Bacillota* and *Actinomycetota,* Mauve=*Thermosulfobacteriota*

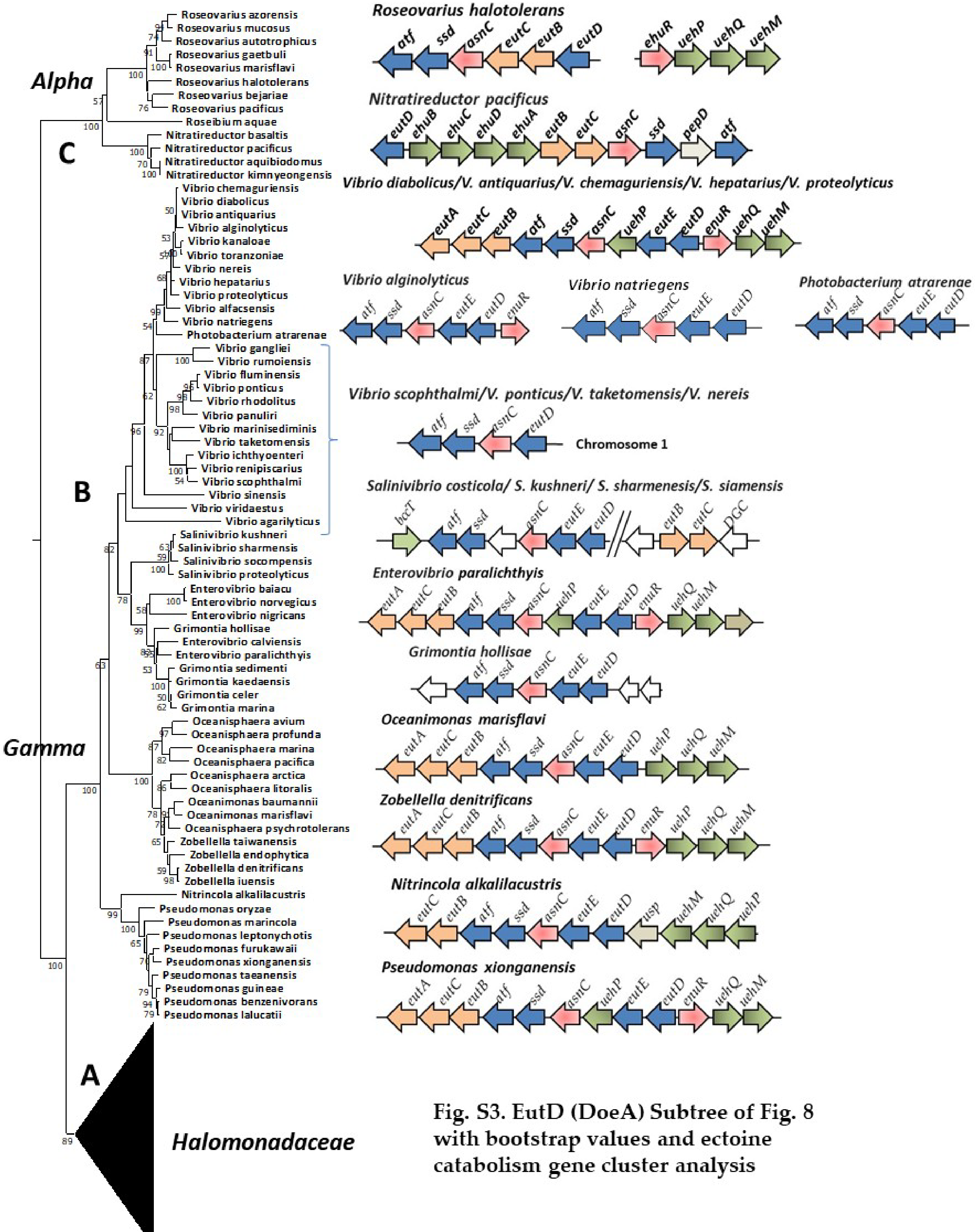

Fig. S3. EutD (DoeA) Subtree of Fig. 8 with bootstrap values and ectoine catabolism gene cluster analysis

Fig. S4. EutD (DoeA) Subtree of Fig. 8 with bootstrap values and ectoine catabolism gene cluster analysis

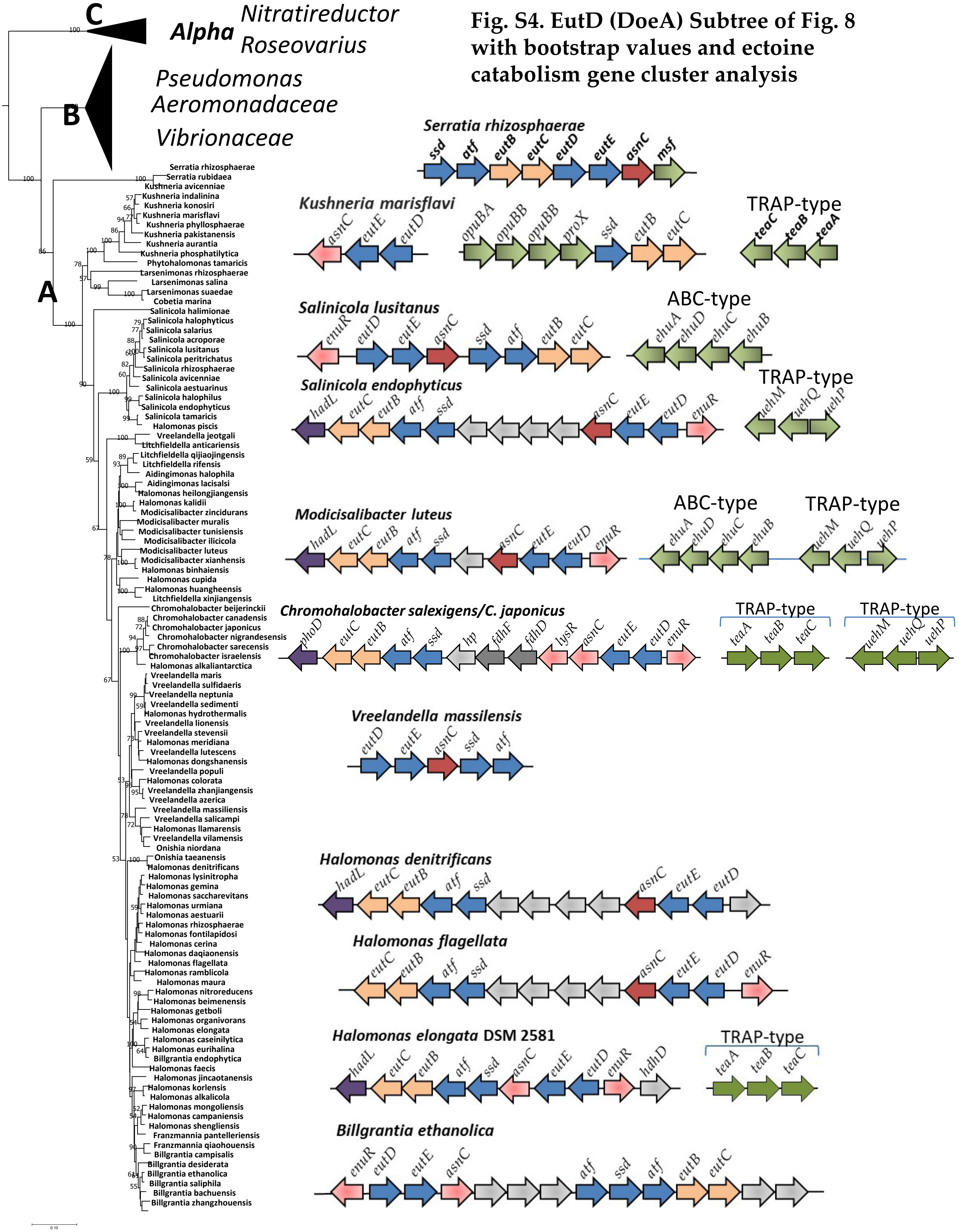

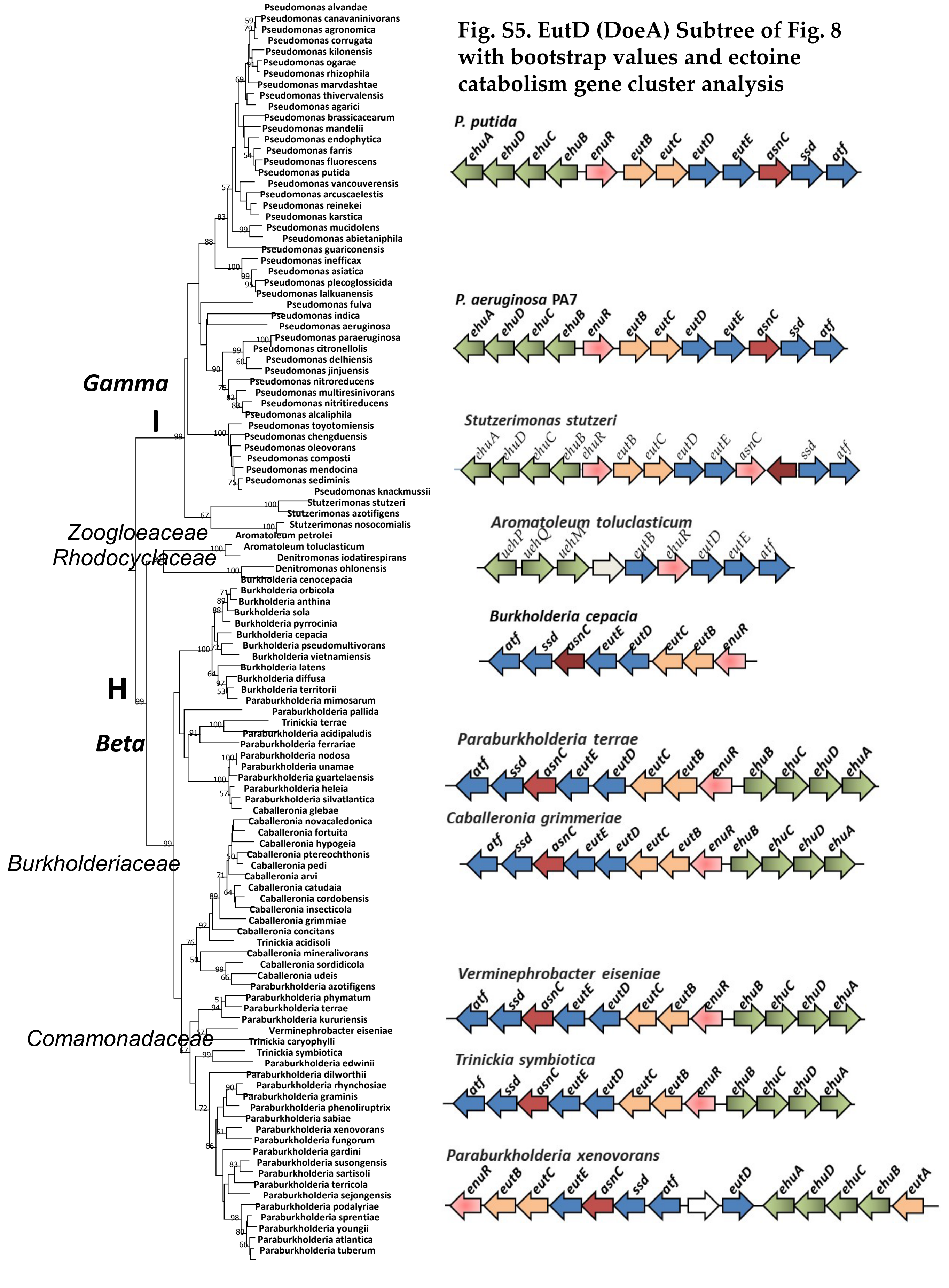

Fig. S5. EutD (DoeA) Subtree of Fig. 8 with bootstrap values and ectoine catabolism gene cluster analysis

Fig. S6. EutD (DoeA) Subtree of Fig. 8 with bootstrap values and ectoine catabolism gene cluster analysis

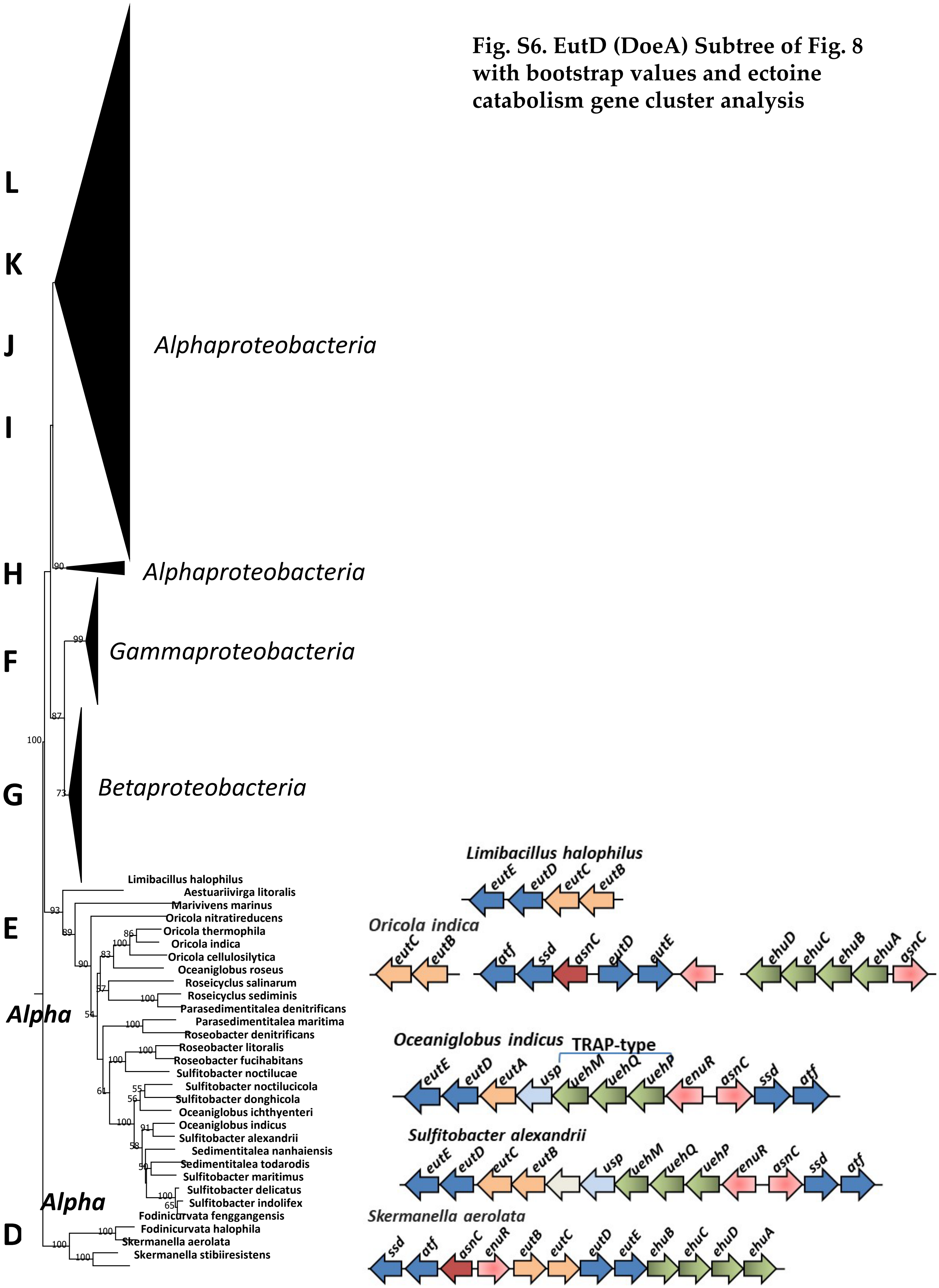

Fig. S7. EutD (DoeA) Subtree of Fig. 8 with bootstrap values and ectoine catabolism gene cluster analysis

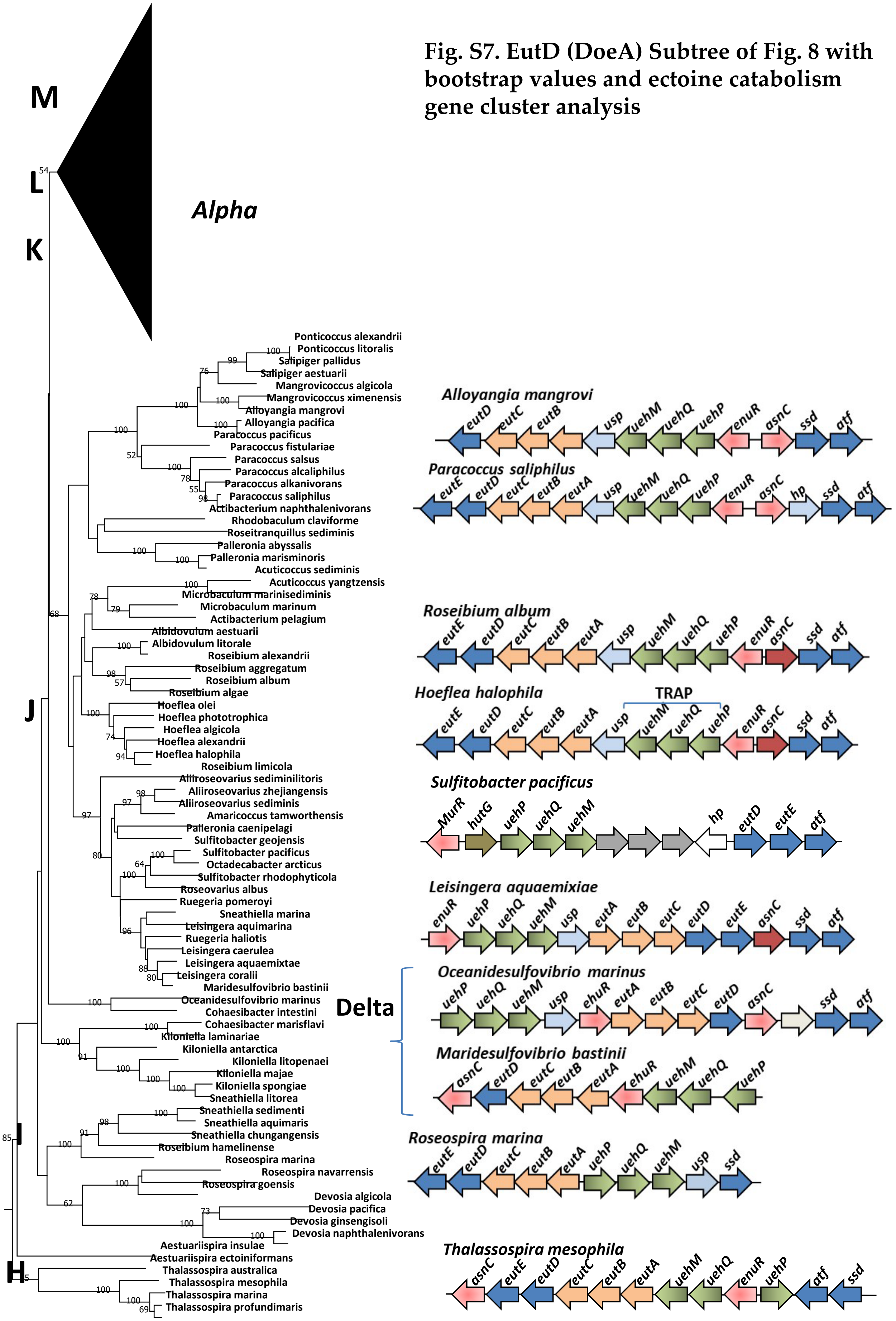

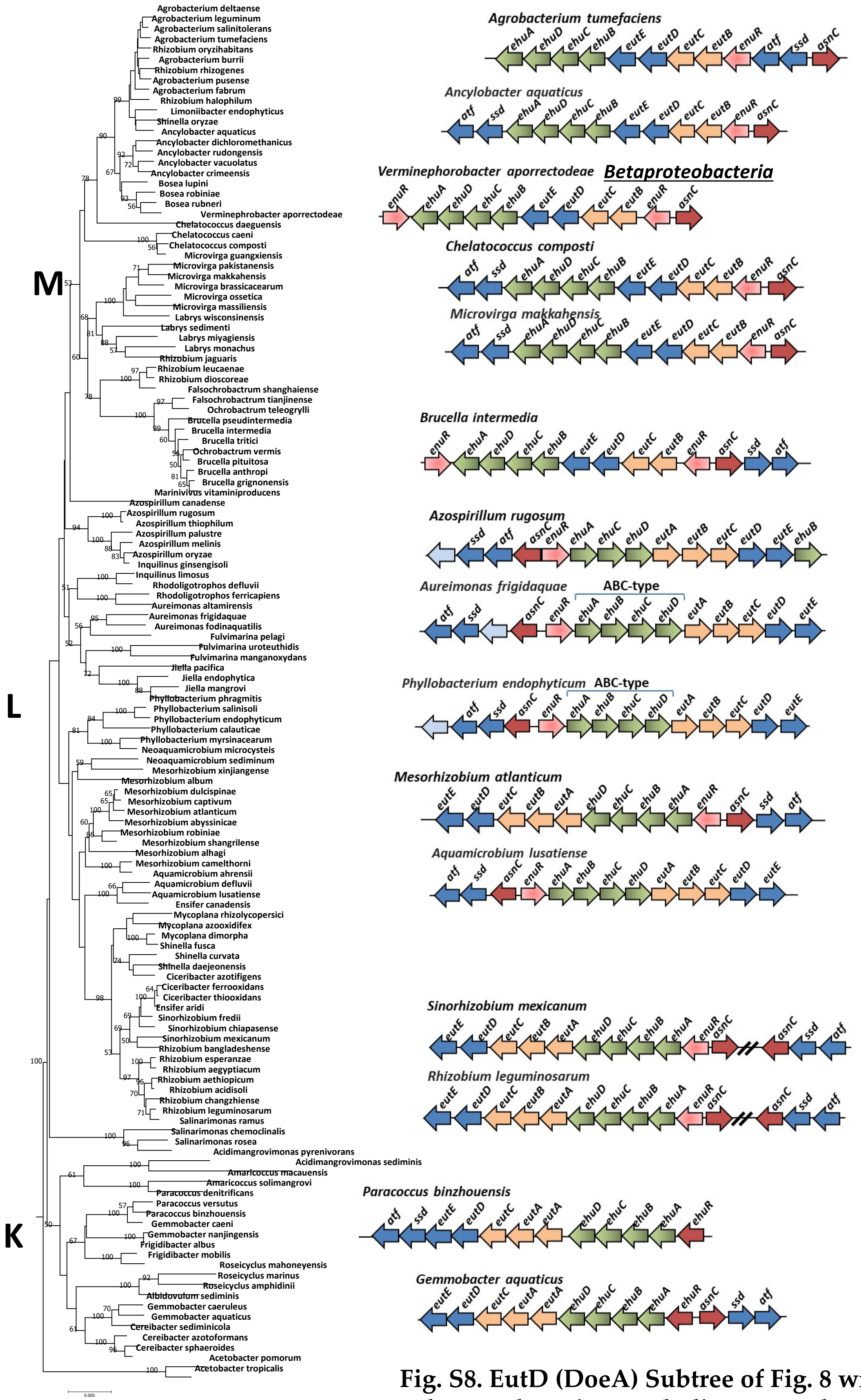

Fig. S8. EutD (DoeA) Subtree of Fig. 8 with bootstrap values and ectoine catabolism gene cluster analysis

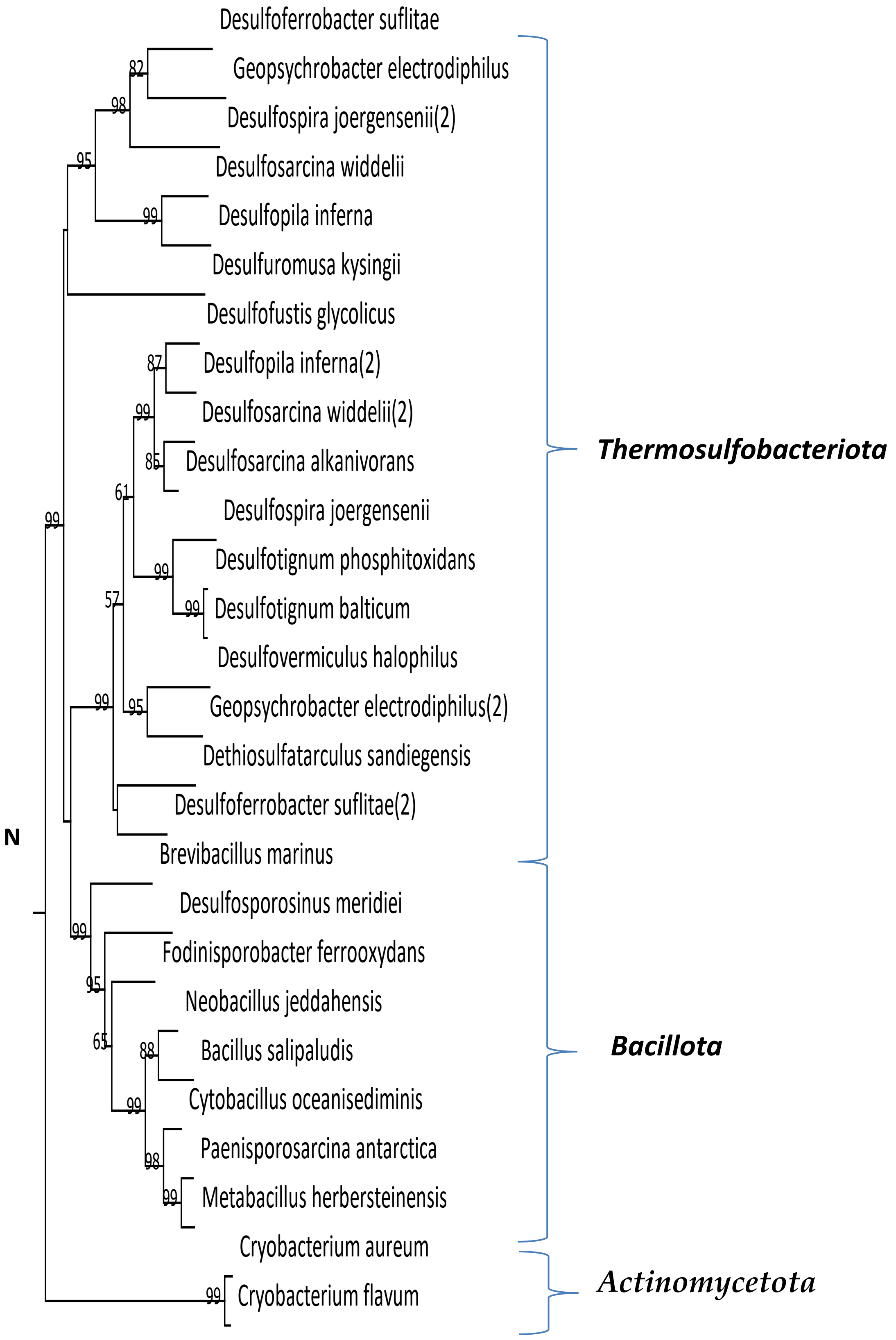

Fig. S9. Evolutionary relationships of ectoine hydrolase EutD (DoeA) from Gram-positive bacteria. Subtree of Fig. 8 with bootstrap values.

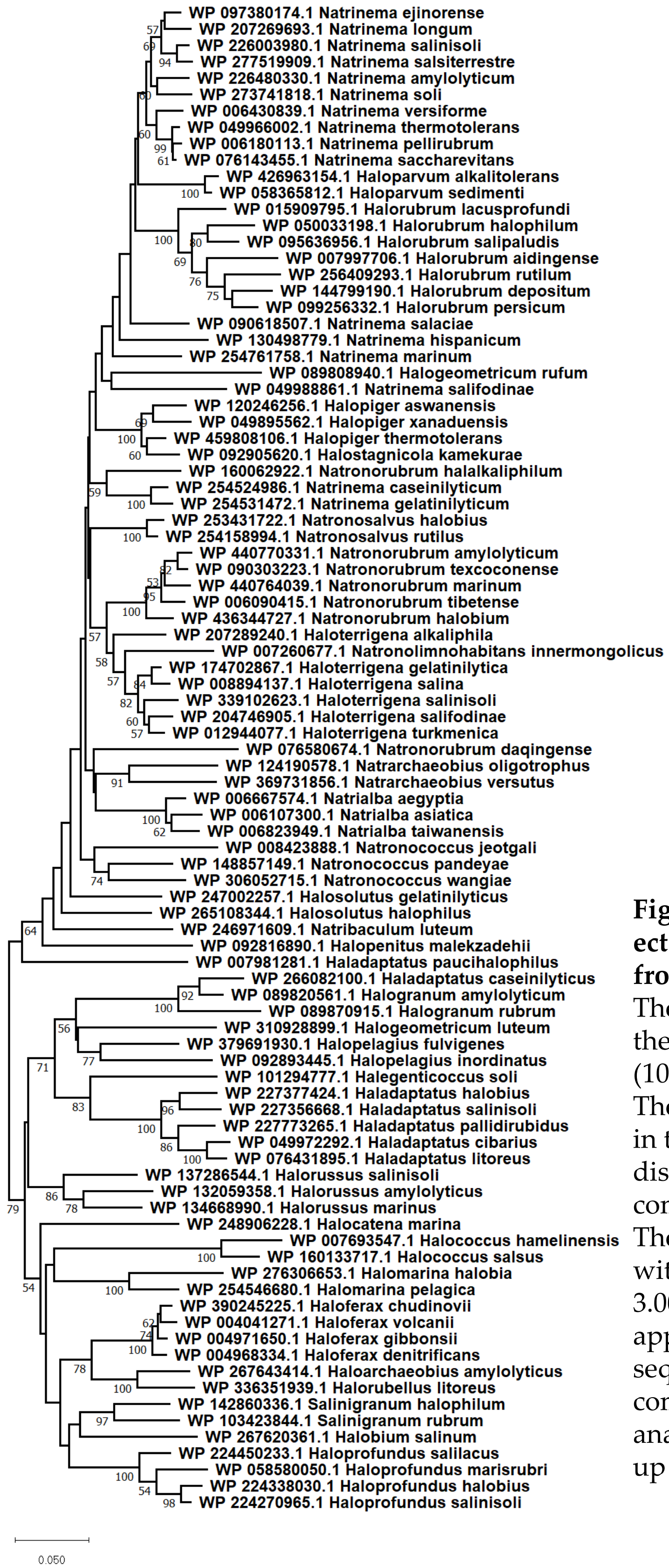

Fig. S10. Evolutionary relationships of ectoine hydrolase EutD (DoeA) from 92 taxa from the domain *Archaea* order *Halobacteria*. The evolutionary history was inferred using the Neighbor-Joining with the bootstrap test (100 replicates) are shown below the branches. The tree is drawn to scale, with branch lengths in the same units as those of the evolutionary distances used to infer the phylogenetic tree computed using the JTT matrix-based method. The rate variation among sites was modeled with a gamma distribution (shape parameter = 3.00). The pairwise deletion option was applied to all ambiguous positions for each sequence pair resulting in a final data set comprising 393 positions. Evolutionary analyses were conducted in MEGA12 utilizing up to 3 parallel computing threads.

Fig. S11 A. Putative ectoine catabolism region present in Domain *Archaea*, Order *Halobacteria*. A. *Haloprofundus halobius* genome assembly ASM2009783v1
B. *Halomarina pelagica* genome assembly ASM2422831v1

A. *Haloprofundus halobius*

NZ_CP083667.1:173995-174972	minus	ABC transporter permease		LAQ74_RS17775	1 WP_224338024.1	325
NZ_CP083667.1:175047-176759	minus	ABC transporter substrate-binding protein		LAQ74_RS17780	1 WP_224338026.1	570
NZ_CP083667.1:176960-178339	plus	**M28 family peptidase**		LAQ74_RS17785	1 WP_224338028.1	459
NZ_CP083667.1:178495-179673	plus	**M24 family metallopeptidase**	**eutD**	LAQ74_RS17790	1 WP_224338030.1	392
NZ_CP083667.1:179728-180978	plus	glutamate dehydrogenase GdhB	**gdhB**	LAQ74_RS17795	1 WP_224338032.1	416
NZ_CP083667.1:181033-184248	minus	**FAD-binding and (Fe-S)-binding domain-**	**GlcD_Glp(**	LAQ74_RS17800	1 WP_224338034.1	1,071
NZ_CP083667.1:184257-185228	minus	D-2-hydroxyacid dehydrogenase		LAQ74_RS17805	1 WP_224338036.1	323
NZ_CP083667.1:185421-186710	plus	**amidohydrolase (aminopeptidase)**		LAQ74_RS17810	1 WP_224338038.1	429
NZ_CP083667.1:186707-187984	plus	**threonine ammonia-lyase**	**ilvA (eutB**	LAQ74_RS17815	1 WP_224338040.1	425
NZ_CP083667.1:188069-189427	minus	**aminotransferase class III-fold pyridoxal**	**AAT**	LAQ74_RS17820	1 WP_224338042.1	452
NZ_CP083667.1:189580-191208	minus	**BCCT family transporter**	**bccT**	LAQ74_RS17825	1 WP_224338044.1	542
NZ_CP083667.1:191766-192413	plus	hypothetical protein		LAQ74_RS17830	1 WP_224338077.1	215
NZ_CP083667.1:192438-192824	minus	Rid family detoxifying hydrolase	**ridA**	LAQ74_RS17835	1 WP_224338046.1	128
NZ_CP083667.1:192897-194276	minus	**aspartate aminotransferase family protei**	**atf**	LAQ74_RS17840	1 WP_224338048.1	459
NZ_CP083667.1:194384-196045	plus	aldehyde ferredoxin oxidoreductase family protein		LAQ74_RS17845	1 WP_224338050.1	553
NZ_CP083667.1:196118-196918	minus	**IclR family transcriptional regulator**		LAQ74_RS17850	1 WP_224338052.1	266
NZ_CP083667.1:198125-199114	minus	PH domain-containing protein		LAQ74_RS17855	1 WP_224338054.1	329
NZ_CP083667.1:199117-200598	minus	PH domain-containing protein		LAQ74_RS17860	1 WP_224338056.1	493

B. *Halomarina pelagica*

NZ_CP100455.1:488258-489361	plus	TrmB family transcriptional regulator		NKI68_RS20840	1 WP_254546666.1	367
NZ_CP100455.1:489922-490392	minus	helix-turn-helix domain-containing protein		NKI68_RS20845	1 WP_254546667.1	156
NZ_CP100455.1:490559-492505	minus	archaea-specific SMC-related protein		NKI68_RS20850	1 WP_254546668.1	648
NZ_CP100455.1:492637-493251	minus	rod-determining factor RdfA	rdfA	NKI68_RS20855	1 WP_254546669.1	204
NZ_CP100455.1:493792-494973	plus	**M24 family metallopeptidase**	pepP	NKI68_RS20860	1 WP_254546670.1	393
NZ_CP100455.1:495059-495856	plus	**IclR family transcriptional regulator**		NKI68_RS20865	1 WP_254546671.1	265
NZ_CP100455.1:495903-497564	minus	**aldehyde ferredoxin oxidoreductase family protein**		NKI68_RS20870	1 WP_254546672.1	553
NZ_CP100455.1:497784-499163	plus	**aspartate aminotransferase family protein**	atf	NKI68_RS20875	1 WP_254546673.1	459
NZ_CP100455.1:499236-499622	plus	**Rid family detoxifying hydrolase**	ridA	NKI68_RS20880	1 WP_254546674.1	128
NZ_CP100455.1:499938-501557	plus	**BCCT family transporter**	bccT	NKI68_RS20885	1 WP_254546675.1	539
NZ_CP100455.1:501646-502998	plus	**aminotransferase class III-fold pyridoxal phosphate-depend**	AAT	NKI68_RS20890	1 WP_254546676.1	450
NZ_CP100455.1:502991-504271	minus	**threonine ammonia-lyase**	ilvA (eutB?)	NKI68_RS20895	1 WP_254546677.1	426
NZ_CP100455.1:504268-505548	minus	**amidohydrolase**		NKI68_RS20900	1 WP_254546678.1	426
NZ_CP100455.1:505661-506632	plus	**D-2-hydroxyacid dehydrogenase**		NKI68_RS20905	1 WP_254546679.1	323
NZ_CP100455.1:506675-507853	minus	**M24 family metallopeptidase**	eutD (doeA)	NKI68_RS20910	1 WP_254546680.1	392
NZ_CP100455.1:508356-509585	plus	**tyrosine-type recombinase/integrase**		NKI68_RS20915	1 WP_254546681.1	409
NZ_CP100455.1:510030-511400	minus	hypothetical protein		NKI68_RS20920	1 WP_254546682.1	456
NZ_CP100455.1:511632-511796	minus	hypothetical protein		NKI68_RS20925	1 WP_254546683.1	54
NZ_CP100455.1:512251-512766	plus	halocyanin domain-containing protein		NKI68_RS20930	1 WP_254546684.1	171
