# Supplementary material for: Deciphering the evolutionary history of ectoine catabolism, a compatible solute utilized by Vibrio diabolicus as an osmoprotectant and a nutrient source
Source: Appl Environ Microbiol. 2026 Apr 20;92(5):e00409-26. doi: 10.1128/aem.00409-26 (PMC13188858; doi:10.1128/aem.00409-26)
Supplement: Supplemental tables — Tables S1 and S2. [file aem.00409-26-s0002.pdf]

Table S1. Ectoine and hydroxyectoine catabolism proteins identified in *Vibrio diabolicus* with homology to *Halomonas elongata* and *Ruegeria pomeroyi.* Names in brackets are nomenclature from *Halomonas elongata*

Vibrio diabolicus	**Protein ID**	**Product**	***Halomonas elongata***	***Rugeria pomeroyi***
**EutA**	WP_134645445.1	Asp/Glu racemase	-	31%
**EutC**	WP_256936410.1	Cyclodeaminase	57%	46%
**EutB**	WP_006741390.1	Hydroxyectoine dehydratase	57%	50%
**Atf (DoeD)**	WP_074190436.1	Aspartate aminotransferase	68%	60%
**Ssd (DoeC)**	WP_243328035.1	NAD-dependent succinate-semialdehyde	63%	52%
**AsnC (DoeX)**	WP_006741393.1	AsnC family regulator	66%	52%
**UehP**	WP_104972313.1	TRAP substrate-binding protein	41% **(TeaA**)	42% **(UehA)**
**EutE (DoeB)**	WP_006741395.1	diaminobutanoate deacetylase	64%	55%
**EutD (DoeA)**	WP_005392731.1	Ectoine hydrolase	63%	62%
**EnuR**	WP_258624098.1	MocR family regulator with PLP-dependent domain	41%	34%
**UehQ**	WP_145472721.1	TRAP transporter small permease	38% (**TeaB**)	44% **(UehB)**
**UehM**	WP_005392736.1	TRAP transporter large permease	64% (**TeaC**)	67% **(UehC)**

Table S2. *Vibrio diabolicus* NaCl tolerance range and growth at different temperatures. Final OD values and maximum growth rates are displayed.

M9 30°C	OD	Growth rate
0	0.14	0.14
1%NaCl	0.56	0.57
2%NaCl	0.64	0.50
3%NaCl	0.63	0.60
4%NaCl	0.64	0.41
5%NaCl	0.66	0.47
6%NaCl	0.56	0.32
7%NaCl	0	0

M9 37°C	OD	Growth rate
0	0	0
1%NaCl	0.49	0.51
2%NaCl	0.70	0.69
3%NaCl	0.72	0.90
4%NaCl	0.83	0.83
5%NaCl	0.67	0.58
6%NaCl	0.76	0.39
7%NaCl	0	0
